# Silicon Extraction from a Diamond Wire Saw Silicon Slurry with Flotation and the Flotation Interface Behavior

**DOI:** 10.3390/molecules29245916

**Published:** 2024-12-15

**Authors:** Lin Zhu, Dandan Wu, Shicong Yang, Keqiang Xie, Kuixian Wei, Wenhui Ma

**Affiliations:** 1 Faculty of Metallurgical and Energy Engineering/National Engineering Research Center of Vacuum Metallurgy, Kunming University of Science and Technology, Kunming 650093, China; 2 State Key Laboratory of Complex Nonferrous Metal Resources Clean Utilization, Kunming University of Science and Technology, Kunming 650093, China; 3 Silicon Industry and Engineering Research Center of Yunnan Province/Silicon Material Industry Research Institution (Innovation Center) of Yunnan Province, Kunming University of Science and Technology, Kunming 650093, China; 4 School of Engineering, Yunnan University, Kunming 650500, China

**Keywords:** diamond wire saw silicon slurry, silicon extraction, flotation kinetics, interface adsorption behavior, DFT calculation

## Abstract

Diamond wire saw silicon slurry (DWSSS) is a waste resource produced during the process of solar-grade silicon wafer preparation with diamond wire sawing. The DWSSS contains 6N grade high-purity silicon and offers a promising resource for high-purity silicon recycling. The current process for silicon extraction recovery from DWSSS presents the disadvantages of lower recovery and secondary pollution. This study focuses on the original DWSSS as the target and proposes flotation for efficiently extracting silicon. The experimental results indicate that the maximal recovery of silicon reached 98.2% under the condition of a dodecylamine (DDA) dosage of 0.6 g·L^−1^ and natural pH conditions within 24 min, and the flotation conforms to the first-order rate model. Moreover, the mechanism of the interface behavior between DWSSS and DDA revealed that DDA is adsorbed on the surface of silicon though adsorption, and the floatability of silicon is improved. The DFT calculation indicates that DDA can be spontaneously adsorbed with the silicon. The present study demonstrates that flotation is an efficient method for extracting silicon from DWSSS and provides an available option for silicon recovery.

## 1. Introduction

Solar photovoltaic (PV) power generation, as a primary source of energy consumption, is poised to play a crucial strategic role in future energy frameworks [1,2,3]. Photovoltaic technology, mainly based on crystalline silicon solar cells [4,5], has developed rapidly in recent years with the continuous decrease in the cost of PV power [6,7]. Solar crystalline silicon cells have undoubtedly become a promising new energy material [8,9,10]. This has led to an increased demand for crystalline silicon and a corresponding waste rise from solar cell production [11,12]. The solar PV waste recovery is an emerging research topic in the new field of energy conversion [13,14]. The production method for solar-grade crystalline silicon wafer is diamond wire cutting, and approximately 30% of the 6N high-purity silicon is transformed into a waste liquid known as diamond wire saw silicon slurry (DWSSS) during the diamond wire cutting process [7,15]. Since the solid silicon contained in DWSSS has a high-purity content, the recycling of DWSSS has become a popular area of research [16,17,18]. Silicon extraction from DWSSS is vital for resource recycling and the sustainable development of the PV silicon industry [19].

The existing industrial recovery process for silicon extraction from DWSSS involves treating the original DWSSS generated through coagulation, sedimentation, pressure filtration, and flame-retardant treatment to obtain a “silicon sludge” filter cake. Then, the “silicon sludge” filter cake is dried, dehydrated, crushed, and compacted to obtain diamond wire saw silicon powder for further purification treatment, ultimately achieving silicon recovery [13]. However, this lengthy recovery process tends to reintroduce metal impurities [20,21], resulting in reduced silicon recovery [22]. As the particle size of the silicon powder and other contaminants from the diamond wire cutting process is in the micron range, recycling is difficult. To achieve the high-value recycling of DWSSS, this study proposes an efficient approach for silicon extraction from DWSSS through flotation [23].

Flotation is an effective and green method for extracting valuable minerals from different ores based on their physical and chemical surface properties [24]. The target minerals can be separated from raw minerals with the addition of flotation reagents thus achieving the recovery of minerals [25,26]. Based on the characteristics of DWSSS, a process for extracting silicon with low moisture content from DWSSS with flotation by using dodecylamine (DDA) as a collector is proposed in the present study. This process has the advantages of high silicon recovery, easy operation, tight process connection, and easy scalability for sustainable silicon recovery.

## 2. Results and Discussion

### 2.1. Effect of Different Collectors on Feasibility of Flotation

**Figure 1:**
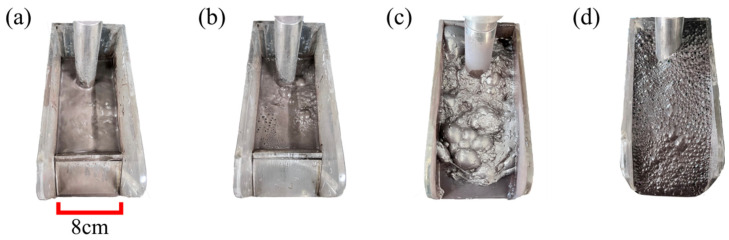
Experimental phenomena of different types of collectors: (**a**) kerosene; (**b**) sodium sulfide; (**c**) DDA; and (**d**) DDA and kerosene.

The capturing effect of different reagents on silicon extraction from DWSSS were compared through silicon capture effect experimentation. The experimental phenomena of kerosene, sodium sulfide, and DDA individually as a flotation reagent for the silicon extraction from DWSSS are displayed in Figure 1a–c, respectively. It can be seen that a large amount of mineral-carrying froth appeared when DDA was used as the collector, indicating that DDA had a significant capturing effect on silicon recovery, so the silicon can be effectively recovered from DWSSS with the addition of DDA. However, when kerosene or Na_2_S was used alone as a flotation reagent, no mineralized foam was observed, indicating that the collector of kerosene and the oxidized ore vulcanizing agent Na_2_S had no significant capture effect on silicon when used alone.

When DDA is used alone as a collector for the flotation silicon from DWSSS, some disadvantages include excessive and sticky mineral-carrying froth, making dehydration challenging and the operation difficult to control. However, a DDA–kerosene mixed collector can alleviate this issue without affecting the silicon recovery [27]. The experimental phenomenon is shown in Figure 1d, where it can be observed that the froth becomes smaller, denser, and thinner with the DDA–kerosene mixed collector [28]. Therefore, the silicon recovery from DWSSS is the highest and the operation is more efficient when DDA and kerosene are used as a combination collector.

### 2.2. Flotation Results of DDA as Collector

**Figure 2:**
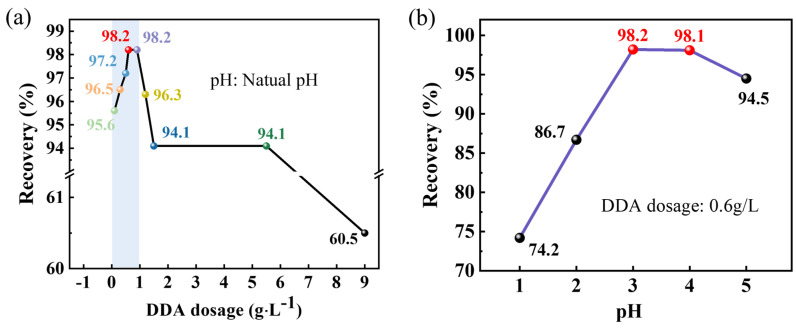
Experimental results of DWSSS flotation: (**a**) recovery of silicon under different DDA dosages; and (**b**) recovery of silicon under different pH levels.

To assess the influence of DDA dosage and pH on silicon recovery, the flotation experiments were conducted under DDA concentrations and pH levels, respectively. The effect of different DDA additions on the recovery of silicon was tested at natural pH. The experimental results are shown in Figure 2a, the blue area is the range of 0-1 g·L^−1^ DDA dosage. where it can be observed that the recovery of silicon concentration increased as the DDA dosage increased within the range of 0.1 g·L^−1^ to 0.6 g·L^−1^, and when the DDA dosage reached 0.6 g·L^−1^, the maximal recovery of silicon reached 98.2%. However, the recovery of silicon began to decline when the DDA dosage reached 1.2 g·L^−1^. The optimal DDA dosage was determined to be 0.6 g·L^−1^, at which the recovery of silicon from DWSSS was the maximum of 98.2%.

To verify the effect of different pH levels on recovery during the flotation experiments, a H_2_SO_4_ or NaOH aqueous solution was used to adjust the pH of the slurry, and the flotation experiments were conducted at different pH values with the DDA dosage of 0.6 g·L^−1^. The experimental results are presented in Figure 2b. The experimental results demonstrate that the prepared slurry is conducive to flotation when the pH level is at the range of 4–5. The original pH of DWSSS is 7–8, and after adding DDA acetate as a collector reagent, the pH of the slurry system is 4–5; this pH is called natural pH. Therefore, the recovery of silicon is the highest with a natural pH of 4–5.

### 2.3. Flotation Kinetics of DWSSS

**Figure 3:**
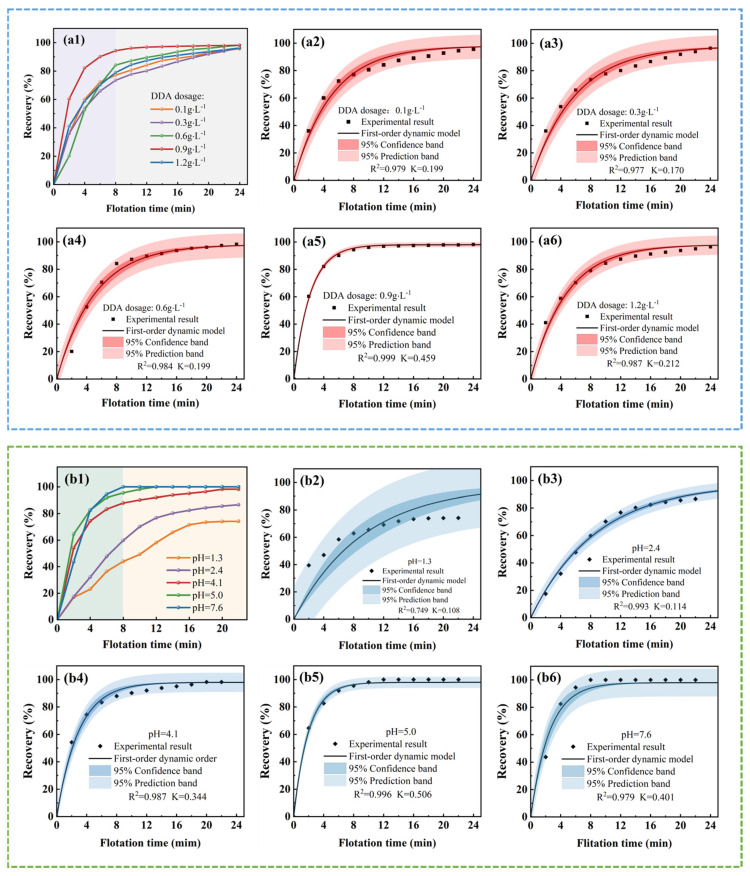
The fitting results of the first-order classical flotation rate model for silicon recovery with different conditions: (**a1**) the recovery of silicon under different DDA dosages; (**a2**–**a6**) the fitting results under different DDA dosages; (**b1**) the recovery of silicon under different pH levels; and (**b2**–**b6**) the fitting results under different pH levels.

The first-order classical flotation rate model was used to fit the flotation kinetics for silicon recovery from DWSSS, and the fitting results are shown in Figure 3. The effects of fitting through the first-order classical kinetics model with different DDA dosages are presented in Figure 3(a1–a6). It can be seen that the R^2^ value reaches 0.98 when the DDA dosage is 0.6 g·L^−1^. The fitting results at different pH values are shown in Figure 3(b1–b6), and it can be seen that the R^2^ value is closest to 1 at a pH level of 5.

The recovery of silicon from DWSSS can be divided into two stages, as illustrated in Figure 3(a1–a6,b1–b6). In the first stage, the flotation rate is fast, and the cumulative recovery of silicon increases rapidly within approximately 8 min. This is because more silicon particles are floating up within the first 8 min, and the added DDA is kept at a relatively high level. The probability of collision between silicon and DDA is high, resulting in a fast flotation rate and a higher recovery [29]. In the second stage, the cumulative recovery of silicon shows a slow upward trend, and the flotation rate decreases significantly after 8 min. This is because the silicon from DWSSS was selected and adsorbed together with a large amount of DDA as the flotation process continues, and the number of silicon particles that can be floated in the slurry significantly decreases, resulting in the decrease in recovery.

**Figure 4:**
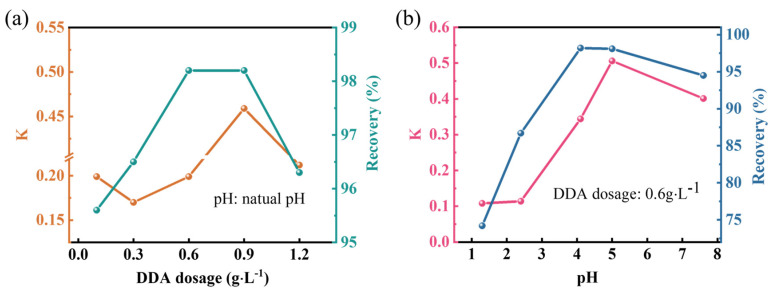
(**a**) Effect of DDA dosage on maximum recovery and flotation rate constant at natural pH; (**b**) effect of pH on maximum recovery and flotation rate constant at DDA dosage of 0.6g·L^−1^.

At natural pH, the effects of different DDA amounts on the maximum recovery and flotation rate constant are shown in Figure 4a. It can be seen that both the maximum recovery and flotation rate constant initially increase and then decrease with the increase in DDA dosage. The recovery reaches a maximum of 98.2% when the DDA dosage is 0.6 g·L^−1^, while the flotation ratio constant reaches a maximum of 0.459 when the DDA dosage is 0.9 g·L^−1^. When the DDA dosage is 0.6g·L^−1^, the effects of different pH values of the slurry system on the maximum recovery and flotation rate constant are shown in Figure 4b. The recovery reaches its maximum of 98.2% at a pH of 4.1; at a pH of 5, the flotation rate constant is 0.506.

### 2.4. Flotation Interface Behavior Analysis

#### 2.4.1. Flotation Interface Behavior Between DDA and Silicon

**Figure 5:**
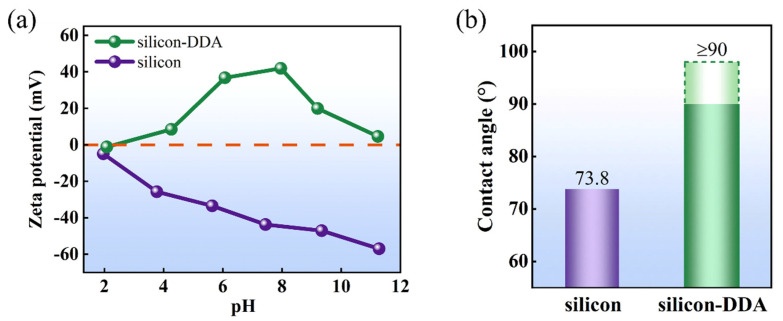
(**a**) Effect of DDA on surface zeta potential of silicon; (**b**) contact angle of silicon before and after DDA addition.

In the study of the flotation interface behavior of DDA on silicon, the dosage of DDA used for preparing samples for analysis was 6 g·L^−1^, and the overall pH range of the slurry was 4–5. The variation in zeta potential at different pH levels before and after the interaction of silicon with DDA is shown in Figure 5a. The zeta potential of silicon decreases with an increasing pH, and the absolute value of the potential increases, indicating that silicon becomes more stable with an increase in pH [30].

The zeta potential of silicon–DDA reached zero at a pH level of approximately 2, indicating a stable flotation system was formed when the pH level was 2. The potential of the silicon–DDA system significantly increases when the pH is 4–8, and the potential change indicates the DDA was adsorbed on the surface of silicon.

The contact angle is a direct indicator of the wetting properties of the mineral surface. The change in the contact angle before and after the interaction of silicon with DDA is presented in Figure 5b. It can be observed that the contact angle of silicon is greater than 90° after the adsorption of DDA. The DDA addition significantly improved the hydrophobicity of the silicon, indicating the increased hydrophobicity of silicon enhances the floatability of silicon in DWSSS. Therefore, the DDA makes the slurry more conducive to the silicon extraction from the DWSSS with flotation.

**Figure 6:**
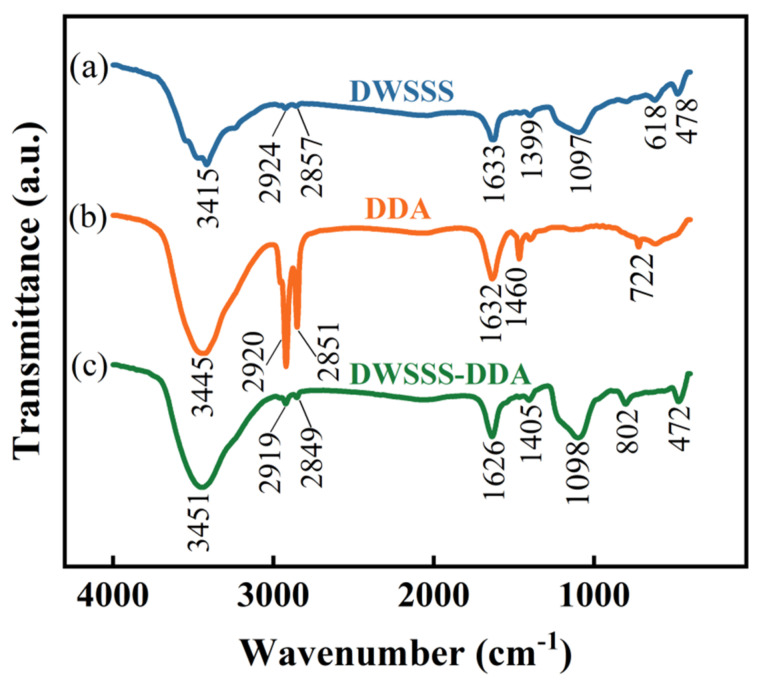
Infrared spectrum results: (**a**) DWSSS; (**b**) DDA; (**c**) DWSSS-DDA.

The infrared spectra of DDA, DWSSS, and DWSSS-DDA are presented in Figure 6. The absorption peak at 3445 cm^−1^, 1632 cm^−1^, and 722 cm^−1^ corresponds to the stretching vibration of N-H, the in-plane bending vibration of N-H, and the out-of-plane bending vibration of N-H [31]. The 2851 cm^−1^ and 2920 cm^−1^ peaks correspond to the symmetric stretching vibration of C-H bonds in the -CH_3_ and -CH_2_- groups, respectively [32]. The peaks at 1460 cm^−1^ correspond to the stretching vibration of C-N bonds. After the adsorption of the DDA, the silicon exhibits an absorption band around 1100 cm^−1^, and the peak corresponds to the anti-symmetric stretching vibration of Si-O-Si bonds [33]. The above results indicate that DDA is adsorbed on the surface of silicon after the addition of DDA.

**Figure 7:**
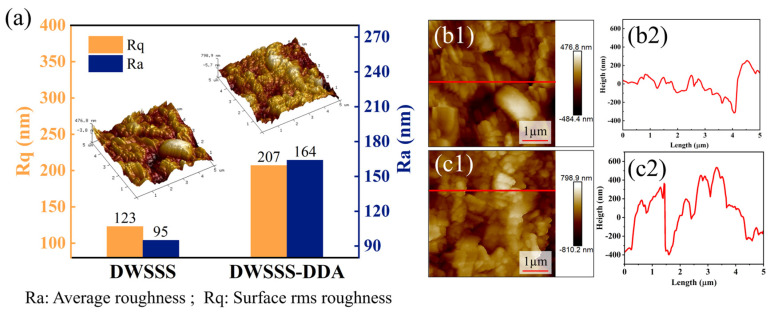
(**a**) Three-dimensional height morphology and roughness changes in DWSSS before and after DDA addition; (**b1,b2**) two-dimensional geometric morphology and cross-sectional height of DWSSS; (**c1,c2**) two-dimensional geometric morphology and cross-sectional height of DWSSS-DDA.

The three-dimensional height morphology, changes in surface roughness value, two-dimensional geometric morphology, and cross-sectional height of DWSSS before and after DDA addition are presented in Figure 7. According to Figure 7a, it can be understood that the Ra and Rq of DWSSS increased with Rq increasing from 123 nm to 207 nm, whereas Ra increased from 95 nm to 164 nm after the addition of DDA. This indicates an increase in surface roughness after silicon adsorption with DDA [34,35,36]. Additionally, Figure 7(b1,b2) shows that the maximum cross-sectional height of DWSSP is about 250 nm, and a smooth surface with a minimum value of around −300 nm appeared in the silicon. On the other hand, Figure 7(c1,c2) shows that the surface of the silicon particle becomes rough after the addition of DDA, where the maximum cross-sectional height of the silicon is approximately 500 nm and a minimum value is approximately −400 nm. This is because the surface of silicon particles becomes rough after DDA adsorption thereby increasing the recovery of silicon, which is consistent with the flotation experiment results.

**Figure 8:**
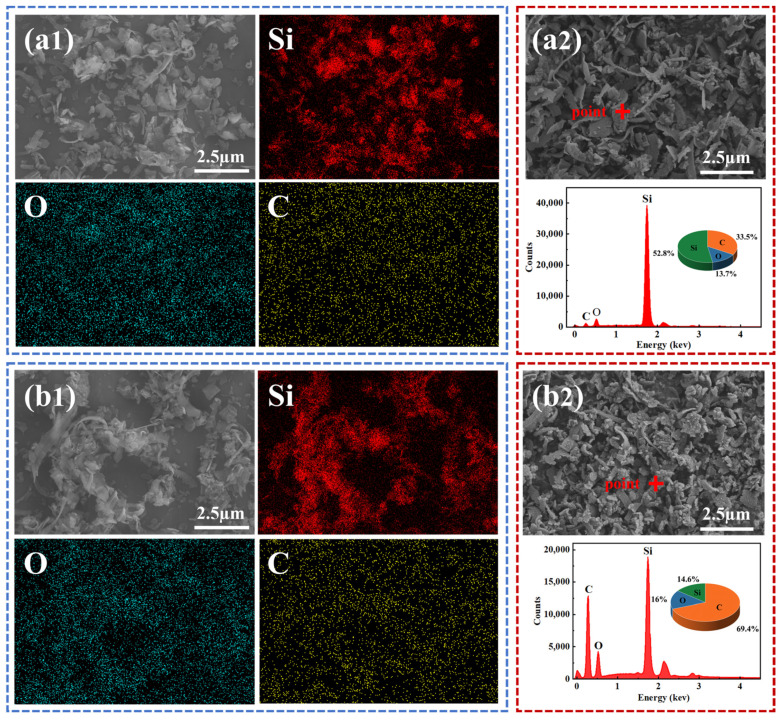
(**a1**) SEM image of DWSSS before DDA addition; (**a2**) EDS result of DWSSS before DDA addition; (**b1**,**b2**) SEM image of DWSSS after DDA addition; (**b2**) EDS result of DWSSS after DDA addition.

The surface morphology of the DWSSS is shown in Figure 8(a1). It can be seen from Figure 8(a2) that the silicon particles in DWSSS and DWSSS–DDA are light gray and mainly composed of Si, O, and C, where the main elements are silicon and carbon, with a silicon content of 52.8% and a carbon content of 33.5%. The surface morphology of the DWSSS–DDA is shown in Figure 8(b1); the carbon content in DWSSS–DDA is 69.4%, and the silicon content is 14.6%, as shown in Figure 8(b2). The significant increase of carbon content indicates that DDA has adsorbed in the silicon surface of the DWSSS after the addition of DDA.

#### 2.4.2. Interface Adsorption Behavior of DDA

**Figure 9:**
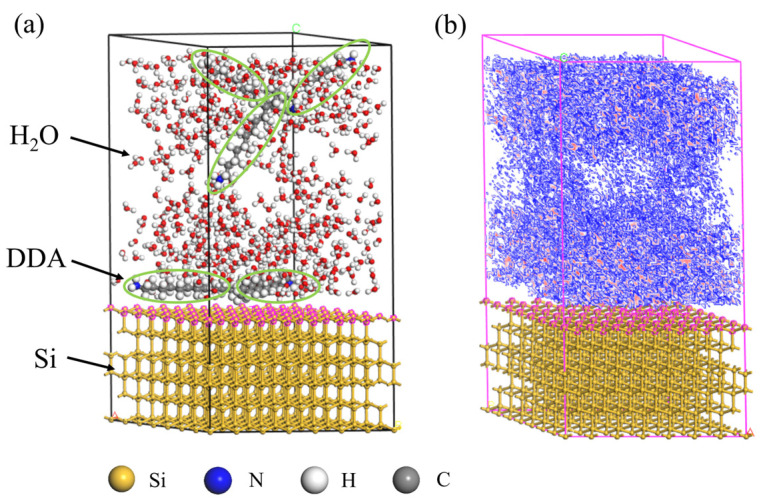
(**a**) Best adsorption position of DDA on Si (111) surface in aqueous solution system; (**b**) density map of adsorption region of DDA on Si (111) surface in aqueous solution.

The Adsorption Locator module is used to locate the adsorption position of DDA molecule on the Si surface. To test the adsorption positions of DDA molecules in an aqueous solution system with Si, an adsorption substrate was established with a Si (111) crystal plane cut to a thickness of 6 Å, a supercell of 7 × 7, and a vacuum layer thickness of 40 Å. The adsorbates consisted of 5 DDA molecules and 500 H_2_O molecules. The calculation results are shown in Figure 9, where Figure 9a presents the optimal adsorption configuration, the green circle is the location of five DDA molecules. It can be seen from the optimal adsorption configuration that DDA, as a macromolecule, has multiple adsorption sites on Si (Top site, Bridge site). The test results indicate that DDA is more inclined to adsorb horizontally on the Si surface. Figure 9b illustrates the most likely adsorption positions of DDA on the Si surface, with the red positions indicating the locations where adsorption is most likely to occur.

**Figure 10:**
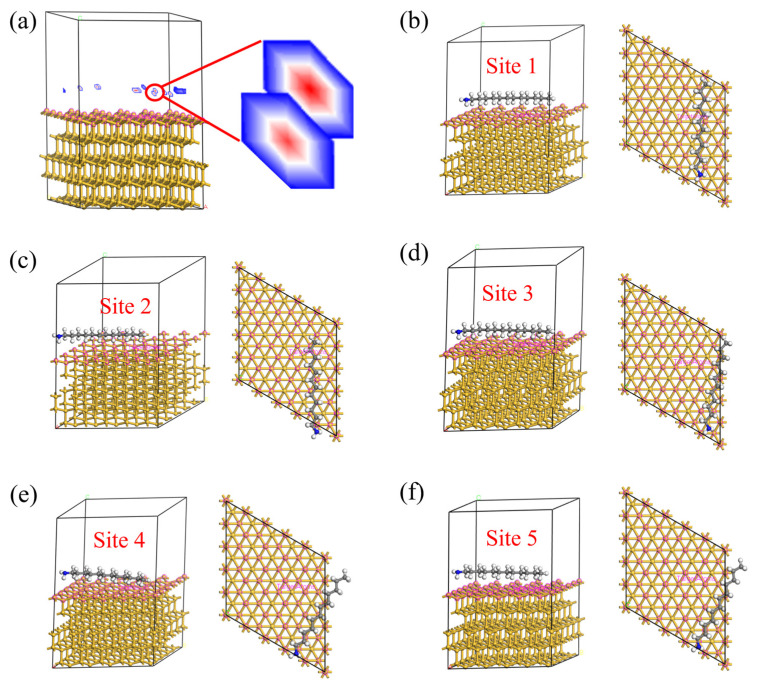
(**a**) Analysis of Si (111) and DDA adsorption sites. Si (111) and DDA possible adsorption position stereogram and top view: (**b**) Site 1, (**c**) Site 2, (**d**) Site 2, (**e**) Site 2, (**f**) Site 2.

In order to further test the adsorption position of DDA on the Si (111) surface, an adsorption substrate is established, and the Si unit cell is cut to obtain the (111) crystal plane, with a surface layer cutting thickness of 4 Å. A supercell of 5 × 5 is created, and the vacuum layer thickness is set to 15 Å. The adsorbate is a single DDA molecule. The calculation results are shown in Figure 10. The analysis of the adsorption positions of Si (111) and DDA is illustrated in Figure 10a, with the most probable adsorption position marked in red. The stereoscopic and top views of the five positions where Si (111) and DDA are most likely to adsorb are shown in Figure 10b–e, and the DDA molecules adsorbed on Si are macromolecules, and there are many adsorption sites (Top site and Bridge site). The adsorption energy of five different adsorption sites is shown in Table 1. It can be seen that the adsorption reaction between Si (111) and DDA can occur spontaneously, the energy is low, and the adsorption model is stable.

**Figure 11:**
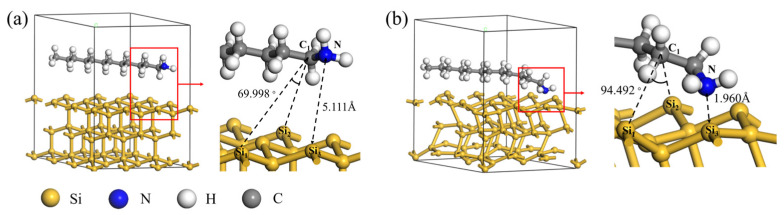
DFT calculation model of DDA on Si (111): (**a**) before geometric optimization; (**b**) after geometric optimization.

Through DFT calculations, the interfacial adsorption behavior between DDA and silicon in the DWSSS is further revealed from a microscopic perspective. The adsorption site of DDA on Si is the bridge site. A Si-DDA model was established with a Si (111) surface layer thickness of 3 Å, a supercell of 3 × 3, and a vacuum layer thickness of 10 Å to explore the adsorption pathway of Si and DDA. The established adsorption model is shown in Figure 11a. The model underwent geometric structure optimization, and the optimized model is shown in Figure 11b. It can be seen that the positions of the atoms in the adsorption model changed after geometric optimization, and the optimized model was used for DFT calculations involving Si atoms and DDA molecules.

**Figure 12:**
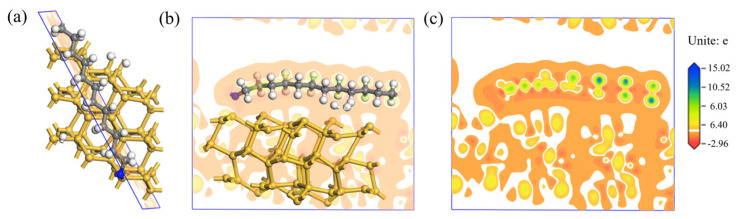
(**a**) Section position of differential charge density diagram of Si-DDA system; (**b**,**c**) differential charge density diagram of Si-DDA system.

After the adsorption process of the Si-DDA system, the differential charge density map was taken from the plane that intersects the maximum number of atoms, as shown in Figure 12a. The differential charge density maps are illustrated in Figure 12b,c. It can be qualitatively analyzed that after DDA is adsorbed on Si, the charge of the H atoms on DDA increases, while the C atoms, N atoms, and the Si atoms lose charge.

The DFT calculation shows the average Mulliken population distribution of each atom in Si-DDA after adsorption, which is listed in Table 2. It can be observed that C gains electrons from the Si (111) surface, while H, N, and Si lose electrons, indicating a transfer of electrons from H, N, and Si atoms to C atoms after adsorption.

**Figure 13:**
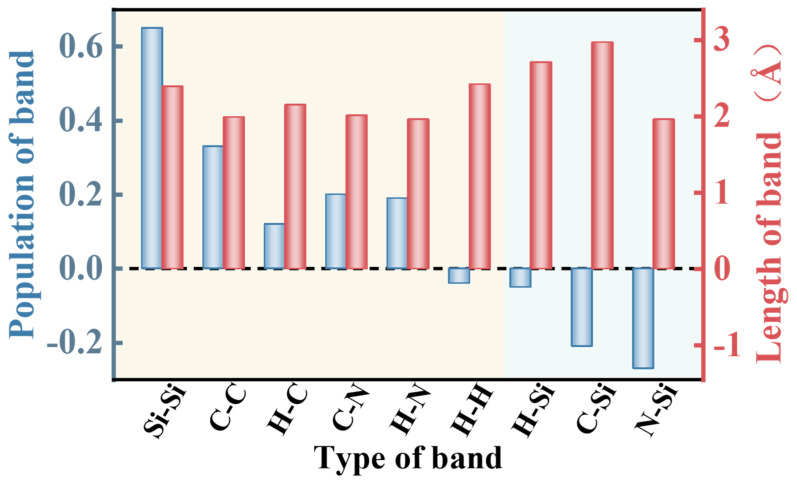
Mulliken population of band in Si (111) surface after DDA adsorption.

The Mulliken population distribution of various chemical bonds after Si and DDA adsorption is shown in Figure 13. It can be observed that the population of Si-Si bonds, C-C bonds, H-C bonds, C-N bonds, and N-Si bonds are all greater than 0. However, the population of H-H bonds, H-Si bonds and C-Si bonds are all less than 0, indicating that H-H bonds, H-Si bonds, and C-Si bonds cannot be formed. By comparing the bond lengths, it can be concluded that the H-N bond with a bond length of 1.96 Å is the shortest among the chemical bonds formed, indicating the strongest covalent bond strength for the H-N bond in DDA.

**Figure 14:**
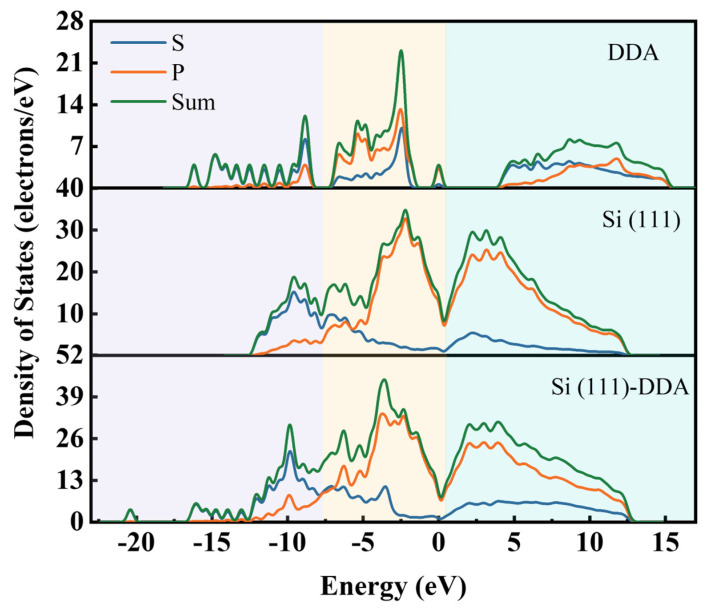
Density of states for DDA, Si (111) surface, and Si-DDA.

The band structure and density of states of the DDA and Si (111) surface before and after DDA adsorption were analyzed to reveal the distribution of the electronic states of Si and DDA during the adsorption process. The density of state distribution is shown in Figure 14. The density of states is divided into three valence bands with the upper valence band located at 0.4~17 eV, the middle valence band located at 0.4~−7.5 eV, and the lower valence band located at −7.5~−23 eV before and after DDA adsorption on the Si (111) surface. The upper valence band at 0.4~17 eV and the middle valence band at 0.4~−7.5 eV are mainly contributed by the P orbitals of the silicon atoms. In contrast, the S orbitals of the DDA atoms mainly contribute to the lower valence band at −7.5~−23 eV.

According to the DFT calculation results, the adsorption energy of Si before the addition of DDA is −8172.65 eV, and the adsorption energy of DDA is −2604.36 eV. The energy of the Si-DDA system after adsorption is −10,780.29 eV. According to Equation (3), the energy difference *ΔE* is −3.28 eV, and the obtained *ΔE* indicated that the adsorption process can spontaneously occur. Furthermore, the calculation results demonstrate that, from a microscopic perspective, DDA can be adsorbed with the silicon of the DWSSS thereby allowing the extraction of silicon from the DWSSS by flotation.

According to the research of the flotation interface behavior between DDA and silicon and the interface adsorption behavior of DDA, DDA can spontaneously adsorb onto silicon in the DWSSS. The roughness and hydrophobicity of the silicon surface were changed after DDA was adsorbed to the silicon surface by flotation, making the silicon floatable thereby enabling the separation of silicon in the DWSSS.

## 3. Materials and Methods

### 3.1. Materials and Reagents

**Figure 15:**
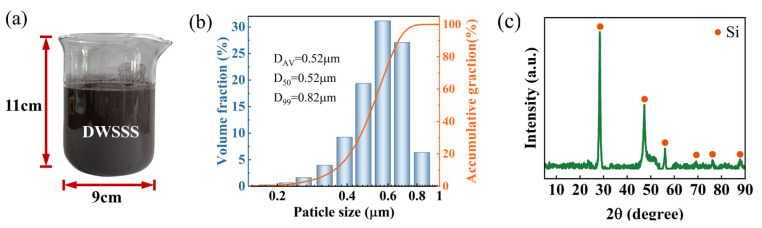
(**a**) Image of original DWSSS; (**b**) particle size distribution of DWSSS; (**c**) XRD phase analysis result of DWSSS.

The DWSSS used in this study was generated during the diamond wire saw cutting process of a solar-grade crystalline silicon wafer. The image of DWSSS is shown in Figure 15a, where the DWSSS is a gray-black suspension with a solid silicon content of 2.11%. The particle size distribution of the DWSSS is presented in Figure 15b, and the silicon particle in DWSSS has an average particle size of 0.52 µm. The XRD analysis of DWSSS for phase composition is demonstrated in Figure 15c, where it is revealed that the phase component is silicon. Since the metal impurity content in DWSSS is usually less than 1000 ppmw [37], this study does not account for the influence of impurities. The non-polar hydrocarbon kerosene with a dosage of 0.2 mL per 400 mL of DWSSS was used as a common collector. The flotation reagent sodium sulfide (Na_2_S) with 0.5 g per 400 mL of DWSSS and cationic collector DDA (CH_3_(CH_2_)_11_NH_2_) with a dosage of 0.4 g per 400 mL of DWSSS were used in the flotation experiment, respectively. The DDA acetate was prepared by DDA and glacial acetic acid in a mass ratio of 1:3. A ratio of 2:3 of DDA to kerosene was mixed and shaken, then heated in a water bath at 50 °C for 10min to prepare a homogeneous solution. The DDA, glacial acetic acid, and sodium sulfide reagents were of analytical grade, while the kerosene was of chemical grade. The amount of flotation reagent used in the preparation of the sample for analysis and detection is 10 times that in the flotation experiment.

### 3.2. Microflotation Experiment

**Figure 16:**
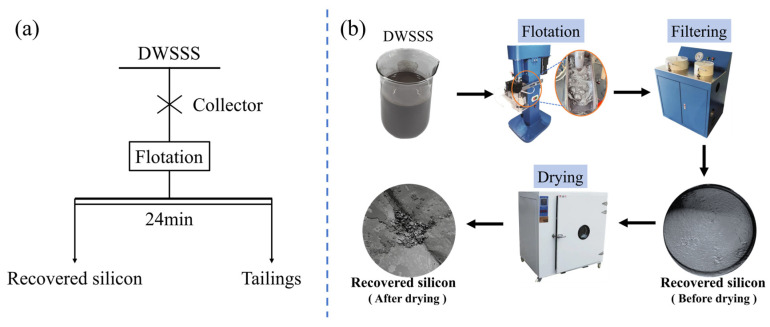
Experimental procedure of flotation: (**a**) flow chart of flotation experiment; (**b**) diagram of flotation process.

The flotation experiment was carried out with an XFD_IV_ flotation machine, and the experimental process is shown in Figure 16a,b. The flotation experimental procedure is as follows: (1) 400 mL of DWSSS is placed into a 500 mL flotation cell, then water is added to the flotation cell to maintain the total volume of the slurry at 500 mL; (2) the impeller speed is set at 1920 r·min^−1^ in the flotation process; (3) the DWSSS is stirred in the flotation cell for 2 min to obtain a homogeneous flotation slurry, then the collector is added to the slurry and stirred for 3 min; (4) air is pumped into the flotation cell at 200–300 L/h; (5) once the air is turned on, twelve two-minute concentrates are collected by scraping the froth every five second, a total flotation time of 24 min; and (6) the recovered silicon concentration and tailings are filtered and then dried at 80 °C until a constant weight is maintained. The recovery of the silicon was calculated by weighing, and the samples were then characterized and analyzed.

The recovery of silicon can be calculated through Equation (1) [38].

(1) $$\varepsilon_{silicon}=\frac{Q_{k}}{Q_{k}+Q_{n}}\times 100\%$$

where Q_k_ (g) is the weight of the silicon concentrate; Q_n_ (g) is the weight of the tailings; and ε_silicon_ (%) is the recovery of the silicon in the DWSSS.

### 3.3. Flotation Kinetics Analysis

The experimental results are fitted by using the first-order classical flotation rate model [39], and the equation can be described through Equation (2).

(2) $$\varepsilon =\varepsilon_{\infty }\left[1-\exp\left(-kt\right)\right]$$

where *ε* (%) is the recovery of the silicon concentrate; *ε_∞_* (%) is the maximum flotation recovery, which is expected to be achieved according to the experimental results, and *ε_∞_* is taken as 98%; *k* is the flotation rate constant; and *t* (min) is the flotation time.

### 3.4. Characterization Methods

The potential was measured by using a zeta potential analyzer (Zeta, Malvern ZEN-3700, United Kingdom). The contact angle was tested by using a surface tension meter (CA, Krüss K100, Germany), through the dynamic capillary penetration method. The chemical bonds on the surface were analyzed by using a Fourier transform infrared spectrometer (FTIR, Bruker ALPHA, Germany). The change in morphology was determined by using an atomic force microscope (AFM, Bruker Dimension Icon, United States). The changes in the surface morphology were observed by using a scanning electron microscope (SEM-EDS, ZEISS Gemini 300, Germany).

### 3.5. DFT Calculation

The adsorption location of DDA on Si was preliminarily calculated by using the Adsorption Locator module of Materials Studio (2023) software. The force field type in the calculation process is COMPASS II, and the fixed energy window is 100 kcal/mol. Density functional calculations were performed on the adsorption of DDA on Si using the CASTEP module. CASTEP simulates the properties of material interfaces and surfaces based on first-principles density functional theory. Utilizing plane wave pseudopotential theory based on total energy, it predicts properties such as lattice parameters and charge density using the number and type of atoms [40]. This study employs the Broyden–Fletcher–Goldfarb–Shanno (BFGS) optimization algorithm and utilizes the generalized gradient approximation (GGA) for exchange-correlation energy, providing a more comprehensive description of charge systems [41]. The Perdew–Burke–Ernzerhof (PBE) functional is applied to describe the exchange-correlation energy [42]. The Tkatchenko–Scheffler method, based on density functional theory (DFT), enhances the accuracy of electronic structure calculations by considering long-range interactions. To optimize traditional van der Waals corrections and improve computational accuracy while maintaining efficiency, the DFT-D3 dispersion correction uses the Tkatchenko–Scheffler method for calculations [43,44].

The crystal structure of Si has the parameters of *a* = *b* = *c* = 5.4307 Å and α = β = γ = 90°. The adsorption energy of DDA on the Si surface could be calculated through Equation (3).

(3) $$\Delta E=E_{Si-DDA}-E_{Si}-E_{DDA}$$

where *ΔE* (eV) denotes the adsorption energy; *E_Si_* (eV) and *E_Si-DDA_* (eV) are the total energy of the Si (111) surface before and after the adsorption, respectively; and *E_DDA_* is the energy of the DDA collector.

## 4. Conclusions

Based on the present study, the following three conclusions can be drawn:

(1) The flotation experiments show that silicon could be efficiently extracted from the DWSSS with flotation, and the maximal recovery of silicon can reach 98.2% with a DDA dosage of 0.6 g·L^−1^ and natural pH within 24 min.
(2) The flotation process follows a first-order kinetics model. When the slurry pH is between 4 and 5, the recovery of silicon is highest when the DDA dosage is 0.6 g·L^−1^, and the flotation rate constant is highest when the DDA dosage is 0.9 g·L^−1^.
(3) The interface and adsorption behavior between the DDA and DWSSS indicate that DDA adsorbs on the silicon surface during the flotation process, modifying the silicon surface and improving hydrophobicity and floatability.

This study proposes an efficient process for silicon extraction from the DWSSS, which has the advantages of high silicon recovery, a short processing cycle, easy operation, and a low possibility of introducing metal impurity to achieve sustainable silicon waste resource recovery.

## Figures and Tables

**Table 1:** Energy results of Si (111) and DDA at different adsorption sites calculated by Adsorption Locator module.

Model	Site 1	Site 2	Site 3	Site 4	Site 5
Total Energy (kcal/mol)	−59.32	−59.12	−58.91	−58.65	−58.45
Adsorption Energy (kcal/mol)	−44.09	−43.89	−43.69	−43.43	−43.22
Adsorption Energy (eV)	−1.90	−1.89	−1.88	−1.87	−1.86

**Table 2:** Average Mulliken population of atoms in Si (111) surface after DDA molecule adsorption.

Type of Atom	Atom Orbit		Total	Charge/e
	S	P		
H	0.37	0.00	10.09	6.81
C	0.64	1.60	26.86	−5.72
N	0.82	2.10	2.90	−0.82
Si	0.70	1.30	108.13	−0.27

## Data Availability

Dataset available on request from the authors.
